# A knowledge translation intervention to enhance clinical application of a virtual reality system in stroke rehabilitation

**DOI:** 10.1186/s12913-016-1807-6

**Published:** 2016-10-06

**Authors:** Danielle Levac, Stephanie M. N. Glegg, Heidi Sveistrup, Heather Colquhoun, Patricia A. Miller, Hillel Finestone, Vincent DePaul, Jocelyn E. Harris, Diana Velikonja

**Affiliations:** 1 Department of Physical Therapy, Movement Sciences and Rehabilitation, Bouve College of Health Sciences, Northeastern University, 407c Robinson Hall, 360 Huntington Ave, Boston, MA 02115 USA; 2 Therapy Department, Sunny Hill Health Centre for Children, 3644 Slocan Street, Vancouver, BC V5M 3E8 Canada; 3 School of Rehabilitation Sciences, Faculty of Health Sciences, University of Ottawa, 200 Lees (A121), Ottawa, ON K1S 5S9 Canada; 4 Department of Occupational Science and Occupational Therapy, University of Toronto, 160-500 University Ave Toronto, Ontario, M5G 1V7 Canada; 5 School of Rehabilitation Sciences, Faculty of Health Sciences, McMaster University, Institute of Applied Health Sciences, Room 403, 1400 Main St. West, Hamilton, ON L8S 1C7 Canada; 6 Bruyere Research Institute, Élisabeth Bruyère Hospital, Bruyère Continuing Care, 43 Bruyère Street, Ottawa, ON K1N 5C8 Canada; 7 School of Rehabilitation Therapy, Queen’s University, Louise D. Acton Building, 31 George Street, Kingston, ON K7L 3 N6 Canada; 8 School of Rehabilitation Science, McMaster University, IAHS Building Room 403, 1400 Main Street West, Hamilton, ON L8S 1C7 Canada; 9 Hamilton Health Sciences, Regional Rehabilitation Centre, 300 Wellington St. North, Hamilton, ON Canada; 10 Department of Psychiatry and Behavioural Neurosciences, DeGroote School of Medicine, McMaster Univerity, 12 Main Street West, Hamilton, ON l8S 1C7 Canada

**Keywords:** Knowledge translation, Virtual reality, Rehabilitation, Stroke

## Abstract

**Background:**

Despite increasing evidence for the effectiveness of virtual reality (VR)-based therapy in stroke rehabilitation, few knowledge translation (KT) resources exist to support clinical integration. KT interventions addressing known barriers and facilitators to VR use are required. When environmental barriers to VR integration are less amenable to change, KT interventions can target modifiable barriers related to therapist knowledge and skills.

**Methods:**

A multi-faceted KT intervention was designed and implemented to support physical and occupational therapists in two stroke rehabilitation units in acquiring proficiency with use of the Interactive Exercise Rehabilitation System (IREX; GestureTek). The KT intervention consisted of interactive e-learning modules, hands-on workshops and experiential practice. Evaluation included the Assessing Determinants of Prospective Take Up of Virtual Reality (ADOPT-VR) Instrument and self-report confidence ratings of knowledge and skills pre- and post-study. Usability of the IREX was measured with the System Usability Scale (SUS). A focus group gathered therapist experiences. Frequency of IREX use was recorded for 6 months post-study.

**Results:**

Eleven therapists delivered a total of 107 sessions of VR-based therapy to 34 clients with stroke. On the ADOPT-VR, significant pre-post improvements in therapist perceived behavioral control (*p* = 0.003), self-efficacy (*p* = 0.005) and facilitating conditions (p =0.019) related to VR use were observed. Therapist intention to use VR did not change. Knowledge and skills improved significantly following e-learning completion (*p* = 0.001) and was sustained 6 months post-study. Below average perceived usability of the IREX (19^th^ percentile) was reported. Lack of time was the most frequently reported barrier to VR use. A decrease in frequency of perceived barriers to VR use was not significant (*p* = 0.159). Two therapists used the IREX sparingly in the 6 months following the study. Therapists reported that client motivation to engage with VR facilitated IREX use in practice but that environmental and IREX-specific barriers limited use.

**Conclusions:**

Despite increased knowledge and skills in VR use, the KT intervention did not alter the number of perceived barriers to VR use, intention to use or actual use of VR. Poor perceived system usability had an impact on integration of this particular VR system into clinical practice.

**Electronic supplementary material:**

The online version of this article (doi:10.1186/s12913-016-1807-6) contains supplementary material, which is available to authorized users.

## Background

Although recent reviews have synthesized the mounting evidence for the effectiveness of virtual reality (VR) interventions in stroke rehabilitation [1–4], little is known about the extent and nature of VR use by physical and occupational therapists outside of a research context [5, 6]. VR is defined as any computer hardware and software system that generates simulations of real or imagined environments with which participants interact using their own movements [7, 8]. Key VR characteristics of immersion, feedback, and interactivity within the virtual world [9] are expressed to varying degrees in both rehabilitation-specific systems and off-the-shelf video gaming consoles. A recent survey of Canadian physical therapists (PTs) and occupational therapists (OTs) found that 46 % of respondents had clinical experience with VR, with the Nintendo Wii being the most familiar and accessible system (Glegg SMN, Levac DE, Miller P, Colquhoun H, Wright V: A survey of physical and occupational therapists’ virtual reality use and learning needs, unpublished). Seventy-six percent of respondents were interested in learning more about VR, with top learning needs relating to equipment set up, game familiarity, and matching games to client goals (Glegg SMN, Levac DE, Miller P, Colquhoun H, Wright V: A survey of physical and occupational therapists’ virtual reality use and learning needs, unpublished). Knowledge translation (KT) resources may support clinicians motivated to use VR in developing the competencies required for evidence-based VR application [10]. As such, developing accessible, effective KT resources is one way to promote the sustainable integration of VR systems within stroke rehabilitation settings.

KT resources for PTs and OTs should support clinicians in designing, monitoring, adapting and evaluating VR-based treatment programs [10]. In addition to technical competence in VR system operation, clinicians must make decisions about which systems and games are most appropriate for their clients’ stages of recovery, goals, and physical and cognitive capabilities [10, 11]. Existing frameworks to support decision-making about VR system selection [12, 13], task analyses of a growing list of games [14, 15] and qualitative reports of therapist and client perceptions of VR use in rehabilitation [11, 16–18] are helpful, but best practice recommendations to guide VR implementation are needed. Known barriers to VR use include lack of time, knowledge, skills and resources; technical issues; and client factors, while practical evidence-informed KT resources are proven facilitators [19, 20]. A previous KT study supporting VR integration in two inpatient acquired brain injury rehabilitation settings targeted modifiable barriers and facilitators using a clinical resource manual, mentoring, technical support and training sessions. Changes in self-reported knowledge and skills was reported by participating therapists [20]. The present KT study builds on these results, focusing on strategies for integrating VR-based therapy in stroke rehabilitation using a more multi-faceted approach.

Systematic reviews of KT strategies in rehabilitation indicate that educational strategies are the most commonly used KT intervention [21–23] but that diversity of study outcomes and interventions limits conclusions about which KT interventions are ideally suited to specific contexts [23]. However, active multi-component interventions improve knowledge and practice behaviors of PTs to a greater extent than passive strategies [24] and interventions addressing specific determinants of practice produce small to moderate effects [25]. Online KT resources, defined as “e-learning products that translate evidence-based knowledge to disseminate information that increases awareness, informs clinical practice and/or stimulates practice change” [26] may positively influence self-reported knowledge and skills [27, 28], and are becoming a popular educational method for health professionals [29]. Indeed, e-learning modules and videos were the preferred educational formats of respondents to the Canadian VR use and learning needs survey (Glegg SMN, Levac DE, Miller P, Colquhoun H, Wright V: A survey of physical and occupational therapists’ virtual reality use and learning needs, unpublished).

The purpose of this study was to develop and to evaluate a KT intervention that incorporated an online module and experiential practice to train PTs and OTs in VR implementation for stroke rehabilitation. The KT intervention was built on previously identified support needs and modifiable barriers in therapist knowledge and skills known to influence VR adoption [5, 20, 30–32]. The KT intervention included a component focused on incorporating motor learning strategies into VR-based rehabilitation; these findings are reported elsewhere (Levac DE, et al: Promoting therapists’ use of motor learning strategies during virtual reality-based stroke rehabilitation, in preparation). The objectives of this study that pertain to this article were to: 1) evaluate the impact of the intervention on therapists’ confidence related to VR knowledge and skills and perceptions of facilitators and barriers related to VR use; 2) assess usability of the VR system; 3) obtain therapists’ perspectives about the KT intervention and VR use in practice; and finally 4) measure the frequency of continued VR use following the KT intervention.

## Methods

The KT intervention took place at 2 sites and was evaluated using a pre-post design. Study procedures are also reported in (Levac DE, et al: Promoting therapists’ use of motor learning strategies during virtual reality-based stroke rehabilitation, in preparation).

### VR system

Motion-capture technology enables players to view their mirror image in the virtual environment of the GestureTek Interactive Rehabilitation Exercise (IREX) software platform (www.gesturetekhealth.com, GestureTek, Toronto, ON, Canada). Interaction with the virtual environment is through body movements to participate with games that address multiple upper extremity or full body movement goals, while motivating clients to participate [33].

### Procedures

PTs and OTs were recruited from two rehabilitation centers with inpatient and outpatient stroke rehabilitation units in two distinct urban centers in Ontario, Canada. Each site had acquired the IREX for clinical and/or research purposes, however therapists had not yet been trained on its use. The KT intervention was delivered at each site over 5 months, with a staggered start time 8 months later for site 2.

During the first month, site 1 therapists completed the self-paced e-learning modules outside of work hours, and also participated in face to face hands-on learning, audit and feedback sessions. Over the next 4 months, each therapist identified up to 4 patients with the following criteria: 1) experienced a stroke within the last 12 months, 2) receiving inpatient or outpatient PT and/or OT services targeting motor skills, and 3) sufficient cognitive and physical skills to engage in VR activities (as determined by treating therapist). Therapists completed up to four sessions of VR-based therapy per patient. Therapists were free to choose the length of time they would use the IREX with each client, and were responsible for all technical aspects of IREX usage (i.e., set-up, etc.). Therapists completed the final outcome measures approximately 1 month after their final study VR-based therapy session.

**Fig. 1:**
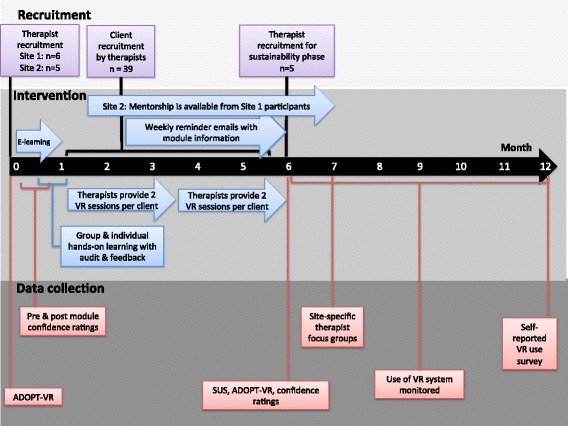
Study procedures, including timing of interventions and outcome measurement

The same process was implemented at site 2. However here therapists had the additional opportunity to seek guidance from a site 1 participant who would act as a clinician-mentor, via email, Skype or phone. KT interventions at each site differed only in this opportunity for mentorship, which was included to explore whether it would impact VR use at either site. At the end of the study, a focus group was conducted at each site to capture therapists’ experiences with VR use. Following the completion of the KT intervention, participants were invited to take part in the sustainability phase, which involved monitoring their use of the VR system over six months; the research team avoided follow-up communication with the therapists during this time (Fig. 1).

### Intervention

The Template for Intervention Description and Replication (TIDieR) Checklist for reporting interventions was used to guide our intervention description below. A similar description can be found in (Levac DE, et al: Promoting therapists’ use of motor learning strategies during virtual reality-based stroke rehabilitation, in preparation).

**Fig. 2:**
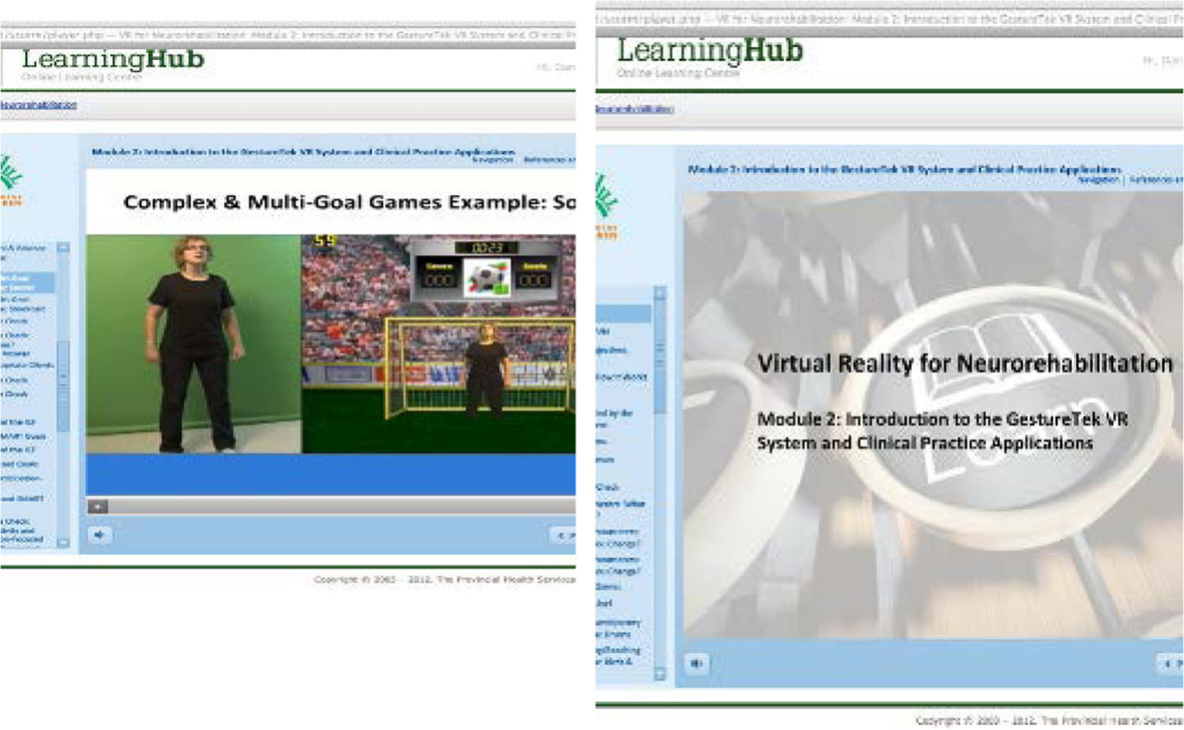
Two screenshots from the Interactive e-learning module

1. *E-learning modules:* Three online modules provided foundational knowledge about clinical VR use. The first module contained information about IREX operation and game characteristics, while the last two modules focused on the application of motor learning strategies to VR-based therapy. Outcomes related to the integration of motor learning strategies are reported in (Levac DE, et al: Promoting therapists’ use of motor learning strategies during virtual reality-based stroke rehabilitation, in preparation). Two co-investigators, with feedback from other authors, developed the modules by integrating their clinical and research experience using VR. Embedded video clips were filmed with consenting client and staff volunteers. Interactive learning activities and knowledge checks required learners to integrate and to demonstrate their knowledge and skills. Based on pilot testing of module usability with therapists at [removed for review], changes were made to the wording of the knowledge checks and material was removed to make the content more manageable. The first module included video clips illustrating IREX game play as illustrated in Fig. 2. Additional file 1 lists the module learning objectives. Therapists completed each e-learning module in approximately two hours. Therapists were also provided with a print manual containing the information presented in the online modules.
2. *Hands-on learning:* Hands-on experience using the IREX was provided during 1 h-long individual session and two group sessions led by the PI and a clinician (MB). These sessions provided additional education and discussion opportunities about VR system operation and trouble-shooting through the use of case scenarios. Case scenarios illustrated ‘typical’ stroke rehabilitation clients and included discussion questions related to game selection, progression of difficulty, and tailoring the task to individual client needs. Feedback about VR skills was provided to participants by the PI during the hands-on learning sessions. Feedback was individualized to each clinicians’ performance and learning needs.
3. *Experiential learning:* Clinicians used the IREX system with up to 4 clients. Feedback from the study investigators about IREX use was available on request via phone, email or in-person support during this period.
4. *Didactic reminders*: Therapists received weekly e-mails from the PI identifying ‘tips’ for VR use from the e-learning modules during the experiential phase.
5. *Mentorship:* Interested clinicians at site 2 were matched with 1 of 3 clinician mentors from site 1 and received biweekly encouragement to contact mentors with questions or support needs. Mentors also reached out periodically to mentees via email.

### Outcomes

#### Knowledge and skills

*Confidence ratings:* Using a seven-point Likert scale, therapists rated their confidence in the knowledge and skills associated with each learning objective before and after completing the e-learning modules as well as at the end of the study. The time between pre- and post-module confidence ratings was not recorded and depended on how long therapists spent completing the module; this ranged from 2 h to 3 days.

### Confidence, barriers and facilitators

*ADOPT-VR:* Therapists completed the Assessing Determinants of Prospective Uptake of Virtual Reality (ADOPT-VR) Instrument pre- and post-study. Based on an extended Theory of Planned Behavior [34]), the ADOPT-VR examines 11 constructs thought to influence VR adoption, including attitudes, self-efficacy, and intention to use VR. The ADOPT-VR has 24 items scored on a nine-point Likert scale (1: Strongly disagree – 9: Strongly agree) as well as nine multiple-response and short-answer questions. Summary scores in 11 categories are reported. Established face and content validity [30], good internal consistency (alpha = 0.876) [35] and responsiveness to change [20] have been demonstrated.

### System usability

*System Usability Scale:* The System Usability Scale (SUS) [36], which has demonstrated reliability, sensitivity and concurrent validity [37], was used to evaluate perspectives of IREX usability at the end of the study. The SUS is a 10 item questionnaire scored on a five-point Likert scale; scores are converted to percentiles. A score of 68 and above is considered above average [38]. Using a traditional school grading scale (from A – F) is recommended to complement the score and convey usability [34].

### Sustainability

Therapists who agreed to continue in the sustainability phase of the study recorded the frequency of their IREX use in the six months post-study completion and provided written responses to two questions about why they had or had not used the IREX.

### Focus groups

The PI conducted a 1.5-h focus group at each site at the end of the study to explore therapist perspectives on participation in the KT intervention, use of the IREX in practice, and experience of integrating motor learning strategies (reported in [32]]. The focus group was audio-recorded and transcribed for analysis.

### Analyses

Non-parametric Wilcoxon signed rank tests evaluated changes between pre- and post-study category scores and frequency counts for the ADOPT-VR as well as changes in confidence ratings at a 0.05 alpha level. Descriptive data were summarized using frequency counts, and means or medians as appropriate. Focus group responses were categorized using content analysis [39] by two investigators.

## Results

### Participant demographics

Six PTs and five OTs participated in the study, enrolling a total of 34 clients. Therapists at site 1 had a mean 19.3 years (SD 8.1) work experience, and therapists at site 2 had a mean of 11.4 (SD 9.4) years work experience. Client mean age was 62.8 (SD 16.4) years at site 1 and 60.1 (SD 15.0) years at site 2. All therapists were novice IREX users. Pre-study, none reported familiarity with any of the IREX games. Following the study, familiarity range was between 4–10 (mean 6.25) games.

### Intervention fidelity

The target client enrolment for each therapist was 4 clients. At site 1, three therapists enrolled four clients, one therapist enrolled three clients, and two therapists enrolled two clients each. At site 2, two therapists enrolled four clients, one therapist enrolled three clients, and two therapists enrolled two clients each. No uptake of the mentoring offered by site 1 clinicians to site 2 clinicians occurred, as measured by participant self-report of phone, email or Skype contact. As such, the KT interventions were identical and results for both sites are presented together.

### Knowledge and skills (confidence ratings)

**Fig. 3:**
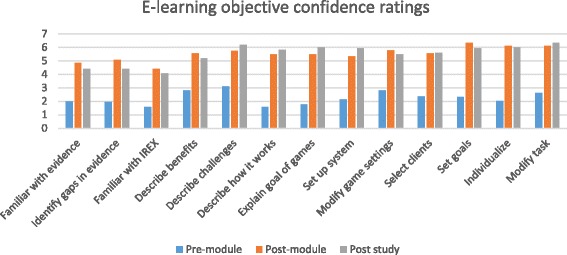
E-learning module objective confidence ratings. Median pre-module, post-module and post-study confidence ratings on each of the e-learning module objectives. Therapists were asked to rate their confidence, on a scale of 1 to 7, in the knowledge and skills associated with each learning objective. Additional file 1 provides the full list of learning objectives

Figure 3 illustrates changes in confidence ratings for each learning objective for the e-learning module. A significant change from pre-module to post-module completion was observed (Z = −3.180, *p* =0.001, median improvement of 3.18 (range 1.46) and this gain was maintained at the end of the study (median decrease of 0.12 points (range 1.24) from post-module completion).

### Knowledge and skills: ADOPT-VR variable outcomes

Significant pre-post improvement in therapists’ perceived behavioral control (Z = −2.945, *p* = 0.003), self-efficacy (Z = −2.802, *p* = 0.005) and facilitating conditions and barriers (Z = −2.352, *p* =0.019) were observed. No other constructs demonstrated significant changes (see Table 1).

**Table 1:** ADOPT-VR pre-post study changes and descriptive data

ADOPT-VR Category	*p*-value	Pre median (range)	Post median (range)
Attitude	0.1	6.67 (4.33–7.67)	6.67 (4–9)
Perceived Usefulness	0.167	6.17 (4.00–8.00)	6.67 (3.67–8.33)
Ease of Use	0.677	5.17 (2.67–7.67)	5.17 (1.67–8)
Compatibility	0.363	4.75 (2–7)	5 (1.5–8)
Social Norms	0.308	5 (1–9)	4.5 (1–6)
Peer Influence	0.194	4.75 (1–7.5)	5 (3–7.5)
Superior Influence	0.403	3 (1–6.5)	3 1.5–7.5)
Perceived Behavioural Control	0.003*	3 (1–6.5)	6.24 (4.5–8)
Self-Efficacy	0.005*	2.5 (1–8)	7 (6–9)
Facilitating Conditions	0.019*	2.75 (1–6)	4.25 (1–7)
Behavioural Intentions	0.21	5.5 (1.67–7.67)	5.33 (1.33–7)

*Significant at *p* < =0.05

### Barriers and facilitators (ADOPT-VR)

Nine therapists indicated in their post-study ADOPT-VR that they intended to continue to use the IREX because of its motivating appeal for clients (e.g. “*I think it is a fun and motivating method of therapy for clients; at times it seems that clients don't even realize it is therapy*”), its potential to add variety to treatment sessions, and its ability to target frequent task repetitions. The two therapists who did not plan on continuing to use the IREX cited lack of time and caseload factors as contributing reasons.

**Fig. 4:**
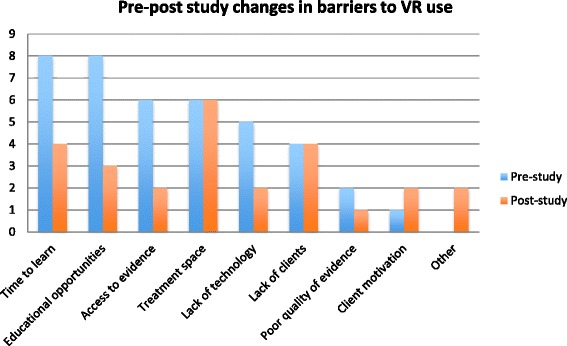
Pre-post study changes in the number of barriers to VR adoption. Frequency count of barriers reported by therapists pre- and post-study

Figure 4 illustrates changes in pre- and post-study frequency counts of reported barriers to IREX use. A decrease in the frequency of reported barriers to IREX use from pre to post study was observed (decrease from 50 to 37 barriers; Z = −1.409, *p* = 0.159). A decrease in the frequency of barriers specifically targeted through this intervention (time to learn about the IREX, access to evidence and educational opportunities; decrease from 22 to 9; Z = 1.1.633, *p* = 0.102) were also observed. In short answer responses that accompany the ratings, therapists described their most significant barriers to IREX use as time, short client length of stay, caseload issues, technical problems with the IREX, and inability to use the system to address OT-related fine-motor goals. For example, one therapist wrote: “*I would use it much more if games included grasp-release and object manipulation.”* Lack of time to use the IREX in a treatment session was the most frequently reported barrier to its use both pre- and post-study. For example, a therapist wrote: “*The timeslot necessary to arrange a decent session is large and the opportunity to set aside that amount of time, plus travel and not have overlap with other patients arriving for treatment is a huge barrier.”* Following the study, therapists described two new barriers on the ADOPT-VR: inappropriate clients and inability to use the IREX to target fine motor goals. Nine therapists reported that adjusting game parameters and resolving technical issues were areas in which they lacked confidence following the study; two therapists did not report any areas of low self-efficacy.

On the post-study ADOPT-VR, seven of the 11 therapists reported greater familiarity with the IREX because of the KT intervention and one therapist reported as a facilitator to IREX use at post-study, having access to patients who were motivated to use VR; three therapists did not describe any facilitators. One therapist commented: *“It would be a nice addition to enhance and add variety to practice for subsets of the patient population. I could identify a few key individuals who would find the program motivational and uplifting*.” No other facilitators were mentioned pre- or post-study.

### System usability

**Fig. 5:**
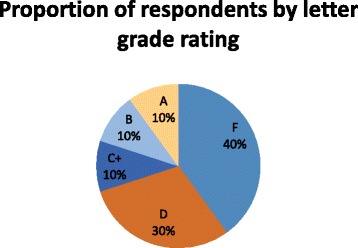
System Usability Scale ratings. An A grade corresponds to a percentile score above 80. A score of 68 is a C grade and scores below 51 correspond to a grade of F

Low perceived usability of the IREX was evidenced by a SUS mean score of 54.25 (19^th^ percentile, below average). Figure 5 shows the proportion of respondents who rated the system by letter grade (from A- F).

### Sustainability

Nine therapists indicated their intention to continue to use the IREX on the ADOPT-VR, and five therapists (three from site 1 and two from site 2) agreed to participate in the sustainability phase of the study. Of the participating therapists, one used the system at site 1 on two occasions and one used the system at site 2 on a single occasion. In written responses following the sustainability phase, these therapists indicated that lack of time, location of the VR room, technical difficulties with the IREX, and lack of appropriate patients were factors limiting their use during the post-study period.

### Focus group

The following three themes were identified regarding the KT intervention and use of the IREX in practice.

#### Benefits and challenges of the KT intervention

Therapists appreciated the combination of self-paced online learning with group and individual practice sessions. The different formats presented in the e-learning modules (e.g. written information, videos and online activities) were also appreciated, although technical issues (e.g. video speed and quality) were an issue for some. Given that the e-learning component took place outside of working hours, therapists reinforced that they would not have undertaken this commitment without the reimbursement provided by the study. Therapists agreed that the opportunity to trial the system/games in person had been helpful; for example, one therapist stated: *“I think it’s a necessity, really, to play around with it yourself, and put yourself in there, and feel the demands and the challenges yourself, will help you decide who to use it with and how to use it more efficiently for sure.”*

However, the fact that their first independent use of the IREX was within the context of a regular treatment session was a challenge for some therapists. This influenced their choice of clients because of the need to balance client safety and expectation of standard treatment with attending to their own (i.e. therapists’) learning. For some therapists, choice of the first client was premised on physical factors, such as independent standing balance, while others sought patients whom they identified as being ‘tech-savvy’. For example, one therapist stated: *“I personally chose a client that I felt was going to be comfortable with the idea of that type of technology. Someone that was really keen on computers…That they would understand that, there might be some glitches and that ….they were giving me [emphasis] an opportunity. They were helping me. That’s what I told them. They were helping me to learn the system, and at the same time, some things that might be helpful for them, too.”* Indeed, therapists expressed that a refresher session during the experiential learning phase would be a useful component of the KT intervention, particularly when more than a few weeks had passed between the initial learning and the therapist’s first use of the IREX with a client. Therapists reported using only the games that they had learned during the KT intervention, implementing frequent repetitions of these games.

#### Client enjoyment was a facilitator of IREX use in practice

Client enjoyment was perceived as a facilitator of IREX use by therapists. For example, one therapist stated: *“[VR] gives a different kind of feedback than what you can do just in the therapy room. It’s a different context. And the fact that it’s a game… one of my patients told me that he didn’t feel like a patient when he was doing it, and he loved that…it was fun, and you know, it made him feel very different from being in a hospital patient, so… there was great value in that.”* Another noted: *“They see it more as a fun and more exciting… I may do the same movement, but it’s more interactive than, let’s say if I’m doing the same repetitive movement without that video. You know, without that interaction with the game….. if you choose the right patients, it gets them excited about possibly the choices or the games that you’re doing.”* Therapists were persistent in encouraging clients who were initially reluctant to try VR-based therapy as they found interest increased with exposure. One therapist noted *“… those people that didn’t necessarily buy in right away, by the end were just like, great, engaged, and motivated, and, you know, really partaking and… right in there.”*

#### Barriers to IREX use in practice

Participants described system and environmental barriers to IREX use in practice. While they were being initially excited to learn a new technology, hardware and software malfunctions that interrupted game play, and poor lighting which interfered with IREX operation were prevalent. Therapists were frustrated when the games did not work. Each site had a contact person who could trouble shoot minor issues related to lighting, but unforeseen software issues with the system itself required maintenance by company representatives, necessitating replacement of equipment and delaying the study at each site by several weeks.

Therapists reported that the IREX games were not always an ideal fit with therapy goals: “*Like, if more games [were available], or just modify it so there’s more for the middle [functioning] person… because I had patients, where I wasn’t comfortable standing them. They were a little bit past sitting and just trying to move, or if they were sitting, like I could work a little bit on their trunk, but there was nothing in between*.” Another said: *“…it wasn’t matching with his goals, and there weren’t really games that were appropriate for his level of activity in his upper extremity.”* Therapists reported that they would be inclined to use a different VR system that could be set up in the standard therapy gym and allow for greater independent patient use. One therapist said: *“I think there’s a value to some of the virtual reality type games, like in the Wii or like the Kinect or whatever. If there was a way to translate that into clients, perhaps in their home setting with family, I think that could be quite interesting for clients…”*

From an environmental perspective, the time required for system set up and to transport a client to the room housing the IREX, which was at some distance from the standard treatment area, was an issue. Therapists in these two settings typically treat multiple clients at once in the large therapy gym, so independent sessions did not fit the standard treatment model. With travel time taken into consideration, less time was available in a session to use the IREX. For example, a therapist stated: *“….it would be the exception that I would do VR. I see some benefit for some patients, but it’s a time–cost ratio. It’s the amount of extra time and work, and what it takes me away from to do VR, versus actually doing something else in the amount of time….”* Finally, the short client lengths of stay at these institutions limited the frequency with which the IREX could be integrated into treatment, since other treatment goals (i.e. discharge readiness) were a priority.

## Discussion

This study targeted therapist knowledge and skills with the goal of increasing clinical uptake of an evidence-based VR system that was already in situ at two clinical sites. Significant increases in self-reports of knowledge and skill were observed and, importantly, sustained over time; however, this change did not translate to increased VR use, although intentions of therapists to continue to use VR were high. The low sample size during the sustainability phase limits the evaluation of long-term behavior change in the presence of high intention to use VR. The study findings strengthen existing knowledge about the facilitators and barriers to VR use with other inpatient populations (21) and add to the evidence base by exploring in greater depth the experience of therapists learning to use VR in practice.

The KT intervention format and content were tailored towards learning needs identified by inpatient clinicians in a previous study [19]. For example, Glegg et al. [20] found that a print educational manual was not well accessed by clinicians working in ABI rehabilitation and suggested e-learning modules, reimbursed training time, and mentoring strategies, all of which were part of this study. The e-learning module and the workshops emphasized information specific to previously identified therapist learning needs [20]. Confidence ratings immediately post-module completion indicated the online learning was effective at improving self-reported knowledge and skills in VR use, with improvements sustained months later. Objective assessments of knowledge and skill change would be valuable adjuncts to future studies to validate this self-report data.

The therapists in this study were novice VR users and they entered the study with positive attitudes towards VR, high perceived usefulness ratings of VR, and strong intention to use the IREX; these ratings remained high at post-test. Because intention to use VR was strong at the study outset, no changes in this construct were expected at post-test. Self-efficacy and perceived behavioral control improved significantly post-study, although 82 % of participants still felt that adjusting game parameters and resolving technical issues were areas in which they lacked confidence. These findings are consistent with previous research [17]. Compatibility, social norms, peer and superior influence constructs of the ADOPT-VR demonstrated no change; these findings are not surprising, given that these variables were not targeted by the KT intervention, and were not anticipated to change. Of note is the absence of change on the perceived ease of use construct, which is in contrast with previous work [20]. A reasonable explanation is the degree of technical difficulty experienced during the current study, as affirmed by the SUS findings; these difficulties were experienced to a greater extent than in previous research [17]. Technical trouble-shooting will likely always be required for VR implementation; having a support person available to manage this aspect is important because these technical skills are outside of most therapists’ skill sets, and the need to trouble-shoot draws time away from both practice opportunities, and client treatment. Indeed, ongoing accessible technical support has been raised as an important element of VR adoption [17], and the low SUS scores suggest that additional support may have been highly valued by these therapists.

Therapists spoke positively of the motivational appeal of the IREX for clients, which is in accordance with previous work describing positive client and therapist perceptions of VR use in practice [5, 11, 17, 40]. However, after gaining experience with the IREX, therapists identified new organizational and client-specific barriers to its use, including a lack of match to treatment goals. Barriers identified through the ADOPT-VR and in the focus groups are consistent with previous work, although treatment delivery method as a barrier (i.e. treating multiple patients at a time) is new to these settings [17]. This may be related to the client population, with the therapists from the previous study working in pediatric and adult ABI rehabilitation likely being more accustomed to 1:1 interventions.

The use of an onsite knowledge broker or clinician ‘expert user’ is a KT strategy with emerging evidence of effectiveness [41, 42]. At site 2, the on-site RA was an expert user with whom clinicians could problem-solve about technical issues. This on-site clinical and technical support, found to be a facilitator of VR use by Glegg et al. [20], was not available at site 1. Glegg et al. [20] observed a spontaneous increase in participating therapists mentoring fellow on-site clinicians in VR use. While informal on-site mentoring was not monitored during this study, the use of cross-site mentoring did not occur despite explicit attempts at facilitation. It may be that our study did not sufficiently facilitate mentor-mentee trust, accessibility, and perceived expertise. Additional efforts to support the establishment of credibility of mentors by mentees may have led to greater success. Future research that explores the optimal timing and delivery of mentoring for VR adoption, as well as therapists’ perspectives about the desirability, benefits and challenges of various mentoring models would build on the knowledge base in this area.

Although the majority of participants indicated they would continue to use the IREX and found VR useful, the five therapists who participated in the sustainability phase did not frequently use the system. While the small sample size and timeframe for follow-up limits generalizability, the reported reasons behind the limited VR use are consistent with the barriers identified in this and previous research [16, 17]. However, despite this observed behavior, a statistically significant increase in facilitating conditions to VR use was found among the larger group of therapists at post-study. Factors such as perceived benefits of VR for clients, the novelty of the intervention, or the support provided through the KT intervention may be responsible for this change, although we did not see an impact on actual use of the VR system. More research is required to determine the relative influence of specific barriers and facilitators on therapists’ behavioral intention and actual behavior related to VR use. To provide greater insight into the sustainability of VR use in the clinical context, and the changing nature or importance of the factors influencing VR use over time, large-scale follow-up studies are needed.

The poor recruitment of clients by therapists during the study was likely related to short client length of stay, some clients declining to participate in the study, and the fact that the IREX was not functioning for several weeks during the study period at each site. Although this problem was accommodated by lengthening the trial, the decreased ‘dosage’ for therapists, in terms of reduced exposure to IREX, likely had an impact on the extent to which therapists were able to integrate clinical knowledge gained during the KT intervention. Such factors may make it more challenging to sustain momentum and behavior change.

Therapists at these sites now have a base level of technical and theoretical knowledge about VR use in practice that they can potentially transfer to other VR systems, though specific learning related to games, equipment set-up and direct clinical application will be required. Barriers unique to this VR system and settings should not be generalized to reflect poorly on the potential for VR implementation as a whole. With respect to the ongoing use of the IREX at these clinical sites, investigators are working with company representatives to resolve the technical issues that arose. Study findings are being discussed with clinical management to strategize around how to address the identified barriers to the use of the technology. Next steps will involve engaging the therapists in a needs assessment to assist them in matching the characteristics of other available VR systems with their specific client and practice setting needs to ensure greater success in future VR implementation efforts.

## Conclusions

This study confirmed previous research demonstrating therapists’ positive attitudes toward VR and its perceived usefulness as a clinical tool for neurorehabilitation. The KT intervention designed to translate knowledge about use of the VR system to therapists in two stroke rehabilitation units was well-received, and successfully incorporated e-learning, experiential learning and reminders to significantly increase self-reported confidence, knowledge and skills in VR use. While these gains were sustained at follow-up, further research is required to examine sustainability of VR use over time in the clinical setting, as well as the feasibility and potential benefits of more structured mentoring models to support VR use. Qualitative findings suggest that system and context-specific barriers merit ongoing attention in order for KT-based interventions that support adoption to be effective. Ongoing training of increasing complexity, paired with interspersed practice in clinical application of the technology, as well as ongoing assessment of therapist support needs are recommended to meet the changing needs of therapists over time.

## Additional file

Additional file 1: Description of data: E-learning module learning objectives Module 1: “Introduction to the GestureTek VR system. (DOCX 14 kb)
