# Supplementary material for: A knowledge translation intervention to enhance clinical application of a virtual reality system in stroke rehabilitation
Source: BMC Health Serv Res. 2016 Oct 6;16:557. doi: 10.1186/s12913-016-1807-6 (PMC5052802; doi:10.1186/s12913-016-1807-6)
Supplement: Additional file 1: — Description of data: E-learning module learning objectives Module 1: “Introduction to the GestureTek VR system. (DOCX 14 kb) [file 12913_2016_1807_MOESM1_ESM.docx]

**Additional File**

E-learning module learning objectives Module 1: “Introduction to the GestureTek VR system”

1. I am familiar with the current state of evidence for virtual reality (VR) in neurorehabilitation.
2. I can identify 3 areas where more research is needed.
3. I am familiar with the current state of evidence specific to the GestureTek system.
4. I can describe 6 potential benefits of integrating VR into my clinical practice.
5. I can describe 3 potential challenges of integrating VR into my clinical practice
6. I can describe how the GestureTek VR system works.
7. I can explain the goal of each of 5 GestureTek games.
8. I can describe the process of setting up the system.
9. I can describe how to modify game software parameters.
10. I can list 5 factors to consider when making decisions about which clients may be appropriate for VR-based therapy.
11. I can set SMART goals for VR-based therapy.
12. I can identify the three aspects of VR-based therapy that can be modified to individualize treatment for different clients.
13. I can provide an example of how to modify each of the task, the virtual environment and the physical environment to improve the therapeutic utility of VR.
14. I can describe 3 factors to consider when delegating VR-based therapy to rehabilitation assistants
